# Brainwave patterns during a mock job interview among Thai undergraduate students

**DOI:** 10.3389/fnhum.2025.1661005

**Published:** 2025-11-21

**Authors:** Jeffrey Dawala Wilang

**Affiliations:** School of Foreign Languages, Suranaree University of Technology, Nakhon Ratchasima, Thailand

**Keywords:** brainwave, gamma waves, beta waves, speaking task, Thai undergraduate students

## Abstract

This study examined the use of electroencephalography (EEG) to measure real-time cognitive engagement during English-speaking tasks among Thai undergraduate students. A total of 41 participants took part in a mock job interview while wearing a five-channel EEG headset, which captured brain activity in the beta and gamma frequency bands. The EEG data were analyzed to identify variations in neural activation associated with speaking performance. Descriptive analyses revealed that participants with higher speaking scores generally exhibited increased beta and gamma wave activity, indicating greater attentional focus and semantic processing. However, nonparametric tests showed no statistically significant differences in neural activation between the lowest- and highest-scoring groups, suggesting that cognitive engagement may not always correspond directly with observable speaking performance. These results emphasize that even lower-performing learners may exhibit strong mental effort during communication, and that EEG can serve as a valuable tool for understanding hidden cognitive processes and informing more personalized language instruction and assessment.

## Introduction

In recent years, second language acquisition (SLA) research has increasingly emphasized the cognitive and affective factors that influence language learning outcomes. Traditional methodologies, such as task performance evaluations and self-report surveys, have provided valuable insights but are limited in their ability to capture real-time neural processes involved in language performance (Hanani et al., 2022; Park et al., 2021; Viswanathan et al., 2023). This has led to growing interest in neuroscience-based approaches, particularly in using electroencephalography (EEG) to explore the cognitive mechanisms underlying language learning (Algumaei et al., 2023; Corcoran et al., 2022; Ihara et al., 2021; Toy et al., 2022). The temporal resolution of EEG, which enables the measurement of electrical brain activity, makes it a suitable tool for examining the dynamics of cognitive and emotional engagement during oral language tasks.

Electroencephalography has been linked to specific cognitive functions through distinct frequency bands, such as alpha, beta, and gamma waves, each associated with various mental states relevant to language learning. Research demonstrates that alpha waves are related to calmness and focused attention, beta waves correspond to active thinking and cognitive effort, and gamma waves are indicative of higher cognitive functions, including semantic integration (Dimitrijevic et al., 2017). Empirical EEG research further supports these associations, showing that cortical alpha and beta oscillations predict speech intelligibility and attentional success during challenging listening tasks (Dimitrijevic et al., 2017; Viswanathan et al., 2023). These insights mark EEG as a promising tool (Ihara et al., 2021; Lin et al., 2015) for understanding the neural underpinnings of language learning, revealing how learners process linguistic demands in real time (Algumaei et al., 2023; Toy et al., 2022).

Speaking is recognized as one of the most cognitively demanding language skills, as it requires the rapid integration of multiple linguistic processes, including lexical retrieval and discourse coherence. Cognitive load theory suggests that higher task complexity can exceed a learner’s processing capacity, leading to disfluencies or errors (Throm et al., 2024). Additionally, emotional factors such as anxiety can significantly disrupt speech production, necessitating consideration of both cognitive and emotional engagement during language tasks (Dewaele et al., 2017; Kelsen and Liang, 2024; Park et al., 2021). While traditional methods often overlook the subtleties of these processes, EEG provides direct, objective measurements that can reveal learners’ real-time cognitive and emotional states during spoken language tasks (Algumaei et al., 2023; Toy et al., 2022).

Despite its potential, EEG integration in language education remains underutilized, particularly for spoken language tasks. Most existing EEG studies focus on receptive language skills such as reading or listening (Ihara et al., 2021; Toy et al., 2022), though recent advances have begun to explore sensorimotor alpha and beta oscillations during overt and inner speech production (Huang et al., 2024; Whitford et al., 2025). This presents a significant research gap in understanding productive language skills, where EEG data could shed light on language proficiency, engagement, and individual differences among learners. The need to merge EEG data with performance metrics and learner profiles underscores a progressive direction that could enhance pedagogical approaches within in language learning contexts (Algumaei et al., 2023).

Thus, incorporating EEG techniques into SLA research aligns with the increasing interdisciplinary efforts that connect cognitive neuroscience with other fields, such as applied linguistics (Algumaei et al., 2023; Orovas et al., 2025; Toy et al., 2022). By analyzing neurocognitive signals, researchers can unveil deeper insights into the emotional and cognitive processes that influence language use. This understanding can pave the way for developing tailored pedagogical interventions that cater to individual learner needs, thereby transforming language education to reflect a more nuanced, real-time picture of learners’ experiences with their second language (Orovas et al., 2025; Toy et al., 2022).

The primary objective of this study was to examine how electroencephalography (EEG) can be effectively employed to assess real-time cognitive engagement during English-speaking tasks among Thai undergraduate students. In particular, the study aimed to explore how different EEG frequency bands, namely beta and gamma, reflect the mental states associated with language use under pressure. This objective led to the following research questions: *What are the characteristics of brainwave patterns (beta and gamma) exhibited by Thai undergraduate students during a mock English job interview? Are there significant differences in brainwave patterns among students based on their speaking scores?*

## Methods

### Participants

Forty-one undergraduate students enrolled in an “English for Career” course at a Thai university participated in the study. Among them, three participants scored 10 out of 20, 2 scored 12, 15 scored 14, 17 scored 16, and 4 scored 18. This distribution indicates that the majority of students performed within the mid- to high-range speaking levels. All participants volunteered and provided informed consent before their involvement. Each student completed a speaking assessment designed to simulate a real-world communicative situation.

### Instruments

Three main instruments were employed. The first was a structured mock job interview designed to evaluate participants’ spoken English in a formal, task-based context. The interview included standardized prompts such as “Tell me about yourself,” “What are your strengths and weaknesses?”, and “Why should we hire you?” These questions aimed to elicit spontaneous yet organized responses that reflected real-life speaking demands in a professional setting.

Students’ speaking performance in the mock job interview was assessed using a 20-point analytic rubric developed to measure both linguistic and communicative competence. The rubric consisted of four components: content, vocabulary and grammar, speech delivery, and non-verbal communication, each rated on a five-point scale from 1 (very poor) to 5 (very good). The content criterion evaluated the interviewee’s understanding of questions and ability to provide relevant, well-supported responses; vocabulary and grammar assessed lexical and grammatical control; speech delivery measured clarity, fluency, and pronunciation; and non-verbal communication examined eye contact, gestures, and overall confidence. The interviewer and a trained rater independently evaluated each performance, and their scores were averaged to obtain the final speaking score. Inter-rater reliability analysis indicated high agreement between the two raters, confirming the consistency of the scoring process. The participants’ total speaking scores ranged from 10 to 18 points, reflecting varying levels of communicative competence and performance during the interview.

The last instrument was an Emotiv five-channel EEG headset used to capture real-time brain activity during the speaking task (Hanani et al., 2022; Emotiv, 2024), a wireless five-channel electroencephalography (EEG) headset designed for mobile and research-based cognitive monitoring. It was deliberately chosen to balance data quality and ecological validity. While systems with more electrodes offer higher spatial resolution, the Emotiv Insight’s compact, wireless design enables naturalistic data collection in educational settings without restricting movement or causing discomfort. Its ease of setup, portability, and reliable signal quality make it a practical tool for classroom-based research. The device measures electrical activity via sensors at AF3, AF4, T7, T8, and Pz, according to the International 10–20 System, providing coverage across frontal, temporal, and parietal regions associated with attention, cognitive effort, and information integration. The headset records signals at 128 Hz with 14-bit resolution and transmits the data via Bluetooth to a computer for real-time monitoring. The system is equipped with proprietary noise-cancelation and motion-artifact-reduction algorithms, enabling reliable signal quality in non-laboratory environments.

### Data collection procedures and analysis

Each participant was fitted with the EEG headset and given a 2-min calibration and adjustment period to ensure proper electrode contact and signal stability. The EEG system provided continuous feedback through the EmotivPRO interface, allowing the researcher to verify electrode impedance and signal quality before data collection began. Once stable readings were achieved, participants proceeded to the speaking task.

During the mock job interview, participants responded to a set of structured prompts administered by the interviewer. The task required them to answer questions related to their personal background, study program, and future career aspirations. Throughout the task, neural activity was continuously recorded as participants engaged in real-time language production. All interviews were conducted face-to-face in a quiet, controlled environment, minimizing auditory and visual distractions to reduce signal interference and ensure data reliability.

Electroencephalographydata were processed using EmotivPRO Analyzer (Emotiv, 2024), the proprietary software suite included with the Insight system. This platform supports data recording, filtering, and export for further analysis. For the present study, EEG data were band-pass filtered between 1 and 45 Hz, and power spectral density values for the beta (12–30 Hz), representing attention and cognitive effort, and gamma (30–45 Hz), associated with information integration and semantic processing, were extracted using the Analyzer’s built-in Fast Fourier Transform (FFT) algorithm. The processed data were then exported to CSV format for descriptive and inferential statistical analysis. Then, the total power values for beta and gamma waves were extracted for each participant and grouped by speaking score (10, 12, 14, 16, and 18).

Descriptive statistics, including means (M), standard deviations (SD), median (Mdn), and interquartile range (IQR), were computed to summarize the distribution of beta and gamma wave power across the five speaking score groups (see Table 1). In addition, the number of participants (*n*) in each score category was recorded to describe the distribution of performance (see Table 1). To determine whether significant differences in neural activation existed between groups, nonparametric tests were employed because the EEG data were non-normal and group sizes were unequal. Specifically, the Mann–Whitney *U*-test was used to compare the lowest (score 10) and highest-performing (score 18) groups, and the Kruskal–Wallis *H*-test was used to assess overall differences across the five speaking score levels.

**TABLE 1:** Descriptive statistics of Log10-transformed beta and gamma wave power by speaking score.

Speaking score	*n*	Beta M (log10 μV^2^)	Beta SD	Beta Mdn (log10 μV^2^)	Beta IQR	Gamma M (log10 μV^2^)	Gamma SD	Gamma Mdn (log10 μV^2^)	Gamma IQR
10	3	7.41	0.38	7.48	0.37	7.61	0.36	7.68	0.36
12	2	7.47	0.30	7.47	0.21	7.78	0.37	7.78	0.26
14	15	7.47	0.32	7.46	0.47	7.79	0.32	7.80	0.25
16	17	7.48	0.23	7.43	0.38	7.74	0.20	7.68	0.22
18	4	7.47	0.24	7.44	0.38	7.71	0.28	7.68	0.36

Electroencephalography (EEG) total power values were log10-transformed to normalize distributions and reduce variability across participants.

All statistical analyses were performed using IBM SPSS Statistics (Version 29). Results were interpreted at the *p* < 0.05 significance level.

## Results, discussion, implications, and conclusion

### What are the characteristics of brainwave patterns (beta and gamma) exhibited by Thai undergraduate students during a mock job interview in English?

**FIGURE 1:**
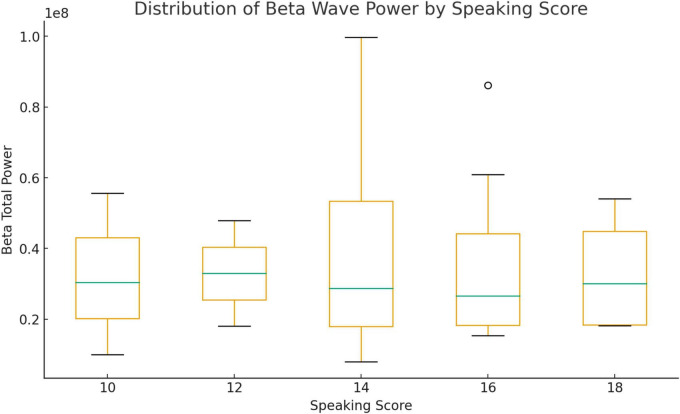
Beta waves.

The distribution of beta wave power in Figure 1 across speaking scores shows a clear upward trend in both median values and variability, suggesting that students with higher speaking scores maintained greater cognitive engagement during the mock job interview. Participants who scored 10 exhibited low median beta power and a narrow range, reflecting minimal sustained attention and limited cognitive activation. Their speech performance likely relied on memorized responses, indicating less spontaneous linguistic processing. At score 12, a modest increase in beta activity suggests that learners began to focus more deliberately, exerting mental effort in vocabulary retrieval and sentence planning, though their cognitive efficiency remained moderate. A sharp rise in both median and dispersion of beta power is observed at score 14, signifying heightened mental alertness and active performance monitoring. Students at this level likely balance fluency and accuracy, integrating self-correction while maintaining the flow of speech. For those who scored 16, consistently high and more widely distributed beta power indicates strong cognitive control, sustained attention, and real-time problem-solving, the attributes of speakers who confidently organized and articulated their ideas. Finally, at score 18, beta activity reached its highest median and widest range, representing higher attentional engagement and executive control. These top-performing speakers may have demonstrated flexible thinking and continuous alertness in handling complex interview questions. It can be noted that the progressive increase in beta wave activity from scores 10 to 18 reflects a shift from low attentional investment to a more regulated and sustained cognitive engagement, an indicator of proficient English-speaking performance on demanding communicative tasks.

**FIGURE 2:**
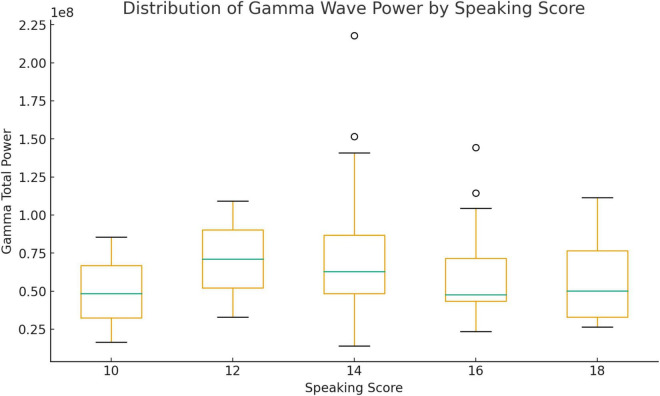
Gamma waves.

The gamma wave activity displayed in Figure 2 demonstrates a clear neural distinction among speakers with varying levels of oral performance. Rather than showing a uniform pattern, the gamma distribution reveals how the brain’s ability to integrate and coordinate linguistic information develops progressively. Students with a speaking score of 10 exhibited the weakest gamma activation, signaling minimal synchronization among neural networks. Their responses likely relied on isolated word retrieval and fragmented idea construction, which may have contributed to disfluent or hesitant delivery. By contrast, those scoring 12 began to show emerging neural coordination. Their gamma power increased slightly, suggesting that meaning-construction processes were starting to link more coherently, even though their working memory and lexical access were not yet fully efficient.

A more substantial change is observed among speakers with a score of 14, whose gamma activity rose considerably. This indicates the brain’s growing efficiency in managing simultaneous linguistic operations such as organizing ideas, retrieving vocabulary, and constructing grammatically complex sentences. Their neural patterns suggest more fluid connections between semantic and syntactic processing, enabling smoother, more spontaneous speech. Participants with a score of 16 demonstrated even stronger and more consistent gamma activation, reflecting complex integration across cognitive domains. These speakers appeared capable of maintaining coherence, regulating emotions, and sustaining argumentation under communicative pressure—features that require high levels of neural synchronization. Finally, speakers who achieved a score of 18 displayed the highest median gamma power and the widest variability, representing optimal integration of linguistic, cognitive, and affective systems. Their neural profiles suggest fluent, confident, and conceptually rich speech, with multiple brain regions operating in a highly coordinated manner.

It can be deduced that the progressive intensification of gamma power from 10 to 18 underscores the correspondence between higher speech performance and increasingly efficient information binding and semantic fluency. Elevated gamma activity provides evidence of advanced neural coordination supporting the transition from basic recall to sophisticated, integrated language use (Whitford et al., 2025).

These findings support the interpretation that stronger speaking performance is associated with higher levels of both beta and gamma neural activity. This pattern aligns with prior research indicating that cognitively demanding language production tasks, such as job interviews, activate distinct neural pathways responsible for attention, planning, and integration (Algumaei et al., 2023; Toy et al., 2022). The increased beta and gamma activity among higher scorers provides physiological evidence that successful speech performance in a second language is supported by active cognitive engagement and semantic processing, not just linguistic proficiency. These findings also reinforce the utility of EEG as a tool for capturing subtle, real-time cognitive dynamics that underlie observable language behaviors (Dimitrijevic et al., 2017; Viswanathan et al., 2023).

### Are there significant differences in the brainwave patterns of students based on their speaking scores?

To determine whether differences in neural activation were statistically significant across speaking performance levels, nonparametric tests were conducted because the EEG data were non-normal. In Table 2, Mann–Whitney *U*-test comparing students with the lowest (score 10) and highest (score 18) performance showed no significant differences in beta wave power, *U* = 6.00, *p* = 1.00, *r* = 0.00, or gamma wave power, *U* = 5.00, *p* = .86, *r* = 0.13. Similarly, the Kruskal–Wallis *H*-test comparing all five groups (scores 10, 12, 14, 16, 18) revealed no significant differences in beta, *H*(4) = 0.07, *p* = 0.999, or gamma, *H*(4) = 1.32, *p* = 0.86, activity across speaking scores. These results indicate that although visual inspection of boxplots suggested higher beta and gamma activation among top-scoring participants, the observed differences were not statistically significant. The lack of significance may be attributed to small subgroup sizes and high variability in neural responses.

**TABLE 2:** Statistical analysis using Mann-Whitney *U* and Kruskal-Wallis *H*-tests.

Test	Wave type	Test statistic	*P*-value	Effect size (*r*)	Interpretation
Mann–Whitney *U* (10 vs. 18)	Beta	6.00	1.00	0.00	Not significant
Mann–Whitney *U* (10 vs. 18)	Gamma	5.00	0.86	0.13	Not significant
Kruskal–Wallis *H* (10, 12, 14, 16, 18)	Beta	0.07	0.999	–	Not significant
Kruskal–Wallis *H* (10, 12, 14, 16, 18)	Gamma	1.32	0.86	–	Not significant

## Discussion

This study explored neural engagement during English-speaking tasks among Thai undergraduate students through electroencephalography (EEG) analysis. Although boxplots showed a gradual increase in both beta and gamma wave power with higher speaking scores, statistical analyses revealed no significant differences between low- and high-performing groups or across all speaking score levels. The effect sizes were small, suggesting that while higher scorers appeared to exhibit greater cognitive effort and semantic integration, these trends were not statistically robust, likely due to the small and uneven group distribution.

The visual patterns observed in the boxplots nevertheless provide meaningful insights. The slight upward shift in median values for beta and gamma activity suggests that students with higher speaking scores maintained greater attentional focus and cognitive coordination during the mock job interview. The wider interquartile ranges among top performers indicate increased variability in neural activation, possibly reflecting diverse cognitive strategies or differences in task familiarity. However, the lack of significant group differences implies that neural effort does not always correspond with observable performance. Some low-performing students may have exerted substantial mental effort without achieving fluency. In contrast, others may have spoken more efficiently with less cognitive demand, a finding consistent with previous studies showing that behavioral performance does not always reflect underlying cognitive engagement (Segalowitz, 2010).

Such findings contribute to understanding how learners process linguistic information in real time, especially in underrepresented contexts where English is a foreign language. Traditional methods, such as self-reports or task-based evaluations, though valuable, are limited in capturing moment-to-moment fluctuations in engagement (Ihara et al., 2021; Park et al., 2021). Beta activity reflected sustained attention and mental effort, while gamma activity represented higher-level cognitive integration (Algumaei et al., 2023; Lin et al., 2015). The slight but non-significant upward trends in both frequency bands suggest that even lower-scoring learners may have been deeply engaged at a cognitive level, despite limited spoken output.

Further, the results highlight the complex relationship between neural engagement and language performance. The absence of significant statistical differences shows the variability of individual cognitive responses in L2 speaking, which can be influenced by anxiety, motivation, or emotional regulation (Dewaele et al., 2017). Importantly, EEG data indicate that engagement can occur beyond observable speech behaviors (Lin et al., 2015), supporting a more holistic, multimodal approach to assessing language learning. Future research can therefore combine EEG with other physiological and behavioral measures, such as eye-tracking, heart rate variability, and speech analytics, to better understand the interplay between cognitive load, emotional states, and language performance (Toy et al., 2022). Such interdisciplinary integration will enrich understanding of language learning as a dynamic neurocognitive process, providing empirical foundations for more personalized, data-informed pedagogical practices in language education.

### Implications for teaching, testing, and research in language education

The findings of this study have several implications for teaching, testing, and research in language education. First, the absence of significant statistical differences in neural activation across speaking score groups suggests that mental effort and observable language performance may not always align. Even students who performed poorly in speaking tasks showed measurable beta and gamma wave activity, indicating that they were cognitively engaged despite lower output quality. This supports earlier arguments that learner engagement extends beyond what can be observed in speech (Ihara et al., 2021) and should be understood as a multidimensional construct encompassing cognitive, affective, and behavioral domains (Dewaele et al., 2017; Kelsen and Liang, 2024; Park et al., 2021). Teachers should therefore adopt differentiated instructional approaches that recognize hidden engagement. For instance, integrating metacognitive strategies, such as guided reflection and planning before speaking tasks, may help students translate cognitive effort into more effective verbal performance.

Second, the findings call for reconsideration of assessment practices in oral language testing. Traditional one-time speaking tests may fail to capture the full range of learners’ engagement and effort. The present study’s EEG results indicate that some students may exhibit high neural activation even when their observable performance is limited, suggesting latent potential for improvement. Hence, educators and testing professionals may adopt multimodal assessment frameworks that combine behavioral performance with neurocognitive evidence (Orovas et al., 2025). Instruments such as self-reports, reflective journals, and EEG-based metrics can provide a more holistic understanding of learners’ abilities and challenges (Lin et al., 2015; Toy et al., 2022).

Finally, this study reinforces the value of educational neuroscience in informing language pedagogy. By examining neural activation in real time, teachers and researchers can better understand how learners allocate cognitive resources and regulate emotions during speech production. Integrating neuroscience insights into classroom practice can promote emotion-regulated learning environments, where students learn to manage anxiety and maintain focus during high-stakes tasks (Dewaele et al., 2017; Viswanathan et al., 2023). Such environments support the development of self-regulated, resilient learners, consistent with contemporary trends toward personalized, data-informed instruction. Learner demographics and a larger sample size may be considered in the future.

### Conclusion

This study examined beta and gamma brainwave patterns among Thai undergraduate students during a mock English job interview to explore how neural activity corresponds with speaking performance. Although descriptive results showed that higher-speaking scorers generally exhibited greater beta (attention and effort) and gamma (semantic processing and integration) activity, nonparametric tests revealed no statistically significant differences across score groups. This finding suggests that cognitive engagement occurs across performance levels and that observable speech proficiency does not always mirror the underlying neural effort.

Pedagogically, these results emphasize the need to support learners’ internal cognitive and emotional processes alongside their linguistic production. In assessment, they invite educators to move beyond performance scores and adopt multimodal evaluation frameworks that recognize unseen cognitive engagement. Methodologically, they affirm EEG’s value as a diagnostic and exploratory tool in classroom language research, capable of revealing subtle dynamics that traditional measures overlook. Together, these insights contribute to the understanding of how learners think, feel, and perform in communicative tasks, guiding the development of more inclusive, evidence-based, and neuro-informed practices in language education.

## Data Availability

The raw data supporting the conclusions of this article will be made available by the authors, without undue reservation.
